# *CCND1* Amplification Contributes to Immunosuppression and Is Associated With a Poor Prognosis to Immune Checkpoint Inhibitors in Solid Tumors

**DOI:** 10.3389/fimmu.2020.01620

**Published:** 2020-08-10

**Authors:** Yu Chen, Yingying Huang, Xuan Gao, Yi Li, Jing Lin, Lizhu Chen, Lianpeng Chang, Gang Chen, Yanfang Guan, Leong Kin Pan, Xuefeng Xia, Zengqing Guo, Jianji Pan, Yaping Xu, Xin Yi, Chuanben Chen

**Affiliations:** ^1^Department of Medical Oncology, Fujian Medical University Cancer Hospital & Fujian Cancer Hospital, Fuzhou, China; ^2^Cancer Bio-immunotherapy Center, Fujian Medical University Cancer Hospital & Fujian Cancer Hospital, Fuzhou, China; ^3^Fujian Provincial Key Laboratory of Translational Cancer Medicine, Fuzhou, China; ^4^Fujian Medical University Cancer Hospital, Fuzhou, China; ^5^Geneplus-Beijing, Beijing, China; ^6^Department of Pathology, Fujian Medical University Cancer Hospital & Fujian Cancer Hospital, Fuzhou, China; ^7^CCIC Group, Kuok Kim (Macao) Medical Center III, Macao, China; ^8^Hui Xian Medical Center, Macao, China; ^9^Department of Radiation Oncology, Fujian Medical University Cancer Hospital & Fujian Cancer Hospital, Fuzhou, China

**Keywords:** cyclin D1 *(CCND1)*, immune checkpoint inhibitors, prognosis, tumor microenvironment, biomarker

## Abstract

*Cyclin D1 (CCND1)* amplification relevant to malignant biological behavior exists in solid tumors. The prevalence and utility of *CCND1* amplification as a biomarker for the clinical response to treatment with immune checkpoint inhibitors (ICIs) are unknown. Our study is a preliminary investigation mainly focused on the predictive function of *CCND1* amplification in the tumor microenvironment (TME) in the aspect of genome and transcriptome. We examined the prevalence of *CCND1* amplification and its potential as a biomarker for the efficacy of ICI therapy for solid tumors using a local database (*n* = 6,536), The Cancer Genome Atlas (TCGA) database (*n* = 10,606), and the Memorial Sloan Kettering Cancer Center (MSKCC) database (*n* = 10,109). Comprehensive profiling was performed to determine the prevalence of *CCND1* amplification and the correlation with the prognosis and the response to ICIs. A *CCND1* amplification occurs in many cancer types and correlates with shorter overall survival and inferior outcomes with ICI therapy. Transcriptomic analysis showed various degrees of immune cell exclusion, including cytotoxic cells, T cells, CD8^+^ T cells, dendritic cells (DCs), and B cells in the TME in a TCGA *CCND1* amplification population. The gene set enrichment analysis suggested that *CCND1* amplification correlates with multiple aggressive, immunosuppressive hallmarks including epithelial–mesenchymal transition, transforming growth factor (TGF)-β signaling, KRAS signaling, phosphoinositide 3-kinase (PI3K)/AKT/mammalian target of rapamycin (mTOR) signaling, p53 pathway, and hypoxia signaling in solid tumors. These findings indicate that *CCND1* amplification may be a key point related to immunosuppression in TME and multiple malignancy hallmarks, and it hinders not only the natural host immune responses but also the efficacy of ICIs.

## Introduction

Immunotherapies targeting immune checkpoints have durable antitumor responses in multiple cancer types, which can contribute to a remarkable improvement in treatment outcomes in a subset of patients with advanced cancer (1). This led to the approval of therapeutic inhibitors of programmed cell death 1 (PD-1) pathway, pembrolizumab, nivolumab, and cemiplimab, and of the programmed cell death ligand 1 (PD-L1) pathway, atezolizumab, durvalumab, and avelumab, by the US Food and Drug Administration (FDA) for the treatment of advanced melanoma, non-small-cell lung cancer, renal cell carcinoma, head and neck squamous cell carcinoma (HNSCC), Hodgkin's lymphoma, squamous cell cancer of the skin, and urothelial bladder cancer. Despite this progress, only a minority of patients within each cancer subtype present with a durable response to immune checkpoint inhibitors (ICIs), and the molecular mechanisms of primary resistance remain incompletely understood (2).

The efficiency of PD-1/PD-L1 inhibitors depends on cancer-specific cytotoxic immune cell activation and infiltration into the tumor microenvironment (TME) (2). Cyclin D1 protein encoded by the *CCND1* gene located on human chromosome 11q13.3 is the critical gatekeeping protein in charge of regulating the transition through the restriction point in the G1 phase to S phase of the cell cycle (3). The *CCND1* gene is considered an oncogene, and it reinforces cell proliferation, growth, angiogenesis, and resistance to chemotherapy and radiotherapy (3, 4). Recently, several studies revealed that *CCND1* amplification associates with a negative response to ICIs. In a study by Saada-Bouzid et al. (5), pretreatment tumor tissue samples from patients who present with hyper-progression after treatments of ICIs were retrospectively detected with next-generation sequencing (NGS). Three out of five patients presented with *CCND1* amplification (5). A retrospective study of melanoma also showed that 30 out of 56 patients with innate resistance to anti-PD-1 therapy presented with *CCND1* amplification (6). Although there are currently few reported cases, the clinical phenomena suggest the potential value of *CCND1* amplification as a biomarker for predicting negative therapeutic effects of ICIs.

We hypothesized that *CCND1* amplification may be associated with poor clinical benefits of ICI therapy through suppressing the antitumor immunity in TME. We mainly focused on the predictive function of CCND1 amplification in the TME in the aspect of genome and transcriptome. In this study, we performed an integrative analysis of The Cancer Genome Atlas (TCGA), Geneplus, and Memorial Sloan Kettering Cancer Center (MSKCC) databases to clarify the frequency of amplification of *CCND1*. Importantly, we aimed to explore whether *CCND1* amplification correlates with a poor response to ICIs in solid tumors, for which the potential mechanism may be correlated with events within the TME.

## Materials and Methods

### Patient Populations

From August 12, 2015, through March 19, 2019, 6,536 tumor tissue samples from 6,536 patients with solid tumors underwent an NGS assay at Geneplus-Beijing (Beijing, China). All procedures were conducted in accordance with the Helsinki Declaration (7) and with approval from the Ethics Committee of Fujian Provincial Cancer Hospital. Written informed consent was obtained from all participants.

### Tissue Processing and DNA Extraction

The germline genomic DNA of peripheral blood lymphocytes and frozen tissue samples was extracted using the DNeasy Blood & Tissue Kit (Qiagen, Hilden, Germany). The formalin-fixed, paraffin-embedded (FFPE) tissue samples were isolated using a commercially available kit (Maxwell® 16 FFPE Plus LEV DNA Purification, Qiagen, Hilden, Germany Kit, catalog: AS1135). The DNA concentration was measured using a Qubit fluorometer and the Qubit dsDNA HS (High Sensitivity) Assay Kit (Invitrogen, Carlsbad, CA, USA). The total DNA yield must be ≥1 μg, while 260/280 and 260/230 ratios are ≥1.8 and 2, respectively.

### Library Preparation, Target Capture, and Next-Generation Sequencing

Sequencing was carried out using Illumina 2 × 75-bp paired-end reads on an Illumina HiSeq 3000 instrument according to the manufacturer's recommendations using the KAPA DNA Library Preparation Kit (Kapa Biosystems, Wilmington, MA, USA). Bar-coded libraries were hybridized to a customized panel of 1,021 genes containing whole exons and selected introns of 288 genes and selected regions of 733 genes (Table S1). The libraries were sequenced to a uniform median depth (>500×) and assessed for somatic variants including single-nucleotide variants (SNVs), small insertions and deletions (InDels), copy number alterations (CNAs), and gene fusions/rearrangements.

### Next-Generation Sequencing Analysis

MuTect2 (version 1.1.4) (8) was employed to identify somatic InDels and SNVs. Contra (v2.0.8) (9) was used to identify CNAs. The CNA was expressed as the ratio of adjusted depth between ctDNA and germline DNA and analyzed using FACETS (10)-with log2ratio thresholds of 0.848 and −0.515 for gain and loss, respectively. Specifically, for the *CCND1* gene, samples with chromosome 11q13.3 alterations were further reviewed for CNAs.

### Data Sources

Solid tumors, which had CNAs identified in the data from 10,606 tumor tissue samples from 10,606 patients with solid tumors in the TCGA, were obtained from the Broad Institute Genomic Data Analysis Center (https://gdac.broadinstitute.org/). The TCGA cohort consisted of adrenocortical cancer (ACC, *n* = 90), bladder urothelial carcinoma (BLCA, *n* = 408), breast invasive carcinoma (BRCA, *n* = 1,080), cervical and endocervical cancer (CESC, *n* = 295), cholangiocarcinoma (CHOL, *n* = 36), colon and rectum adenocarcinoma (COADREAD, *n* = 616), esophageal carcinoma (ESCA, *n* = 184), glioblastoma multiforme (GBM, *n* = 577), HNSCC (*n* = 522), kidney chromophobe carcinoma (KICH, *n* = 66), kidney clear cell carcinoma (KIRC, *n* = 528), kidney papillary cell carcinoma (KIRP, *n* = 288), lower grade glioma (LGG, *n* = 513), liver hepatocellular carcinoma (LIHC, *n* = 370), lung adenocarcinoma (LUAD, *n* = 516), lung squamous cell carcinoma (LUSC, *n* = 501), mesothelioma (MESO, *n* = 87), ovarian serous cystadenocarcinoma (OV, *n* = 579), pancreatic adenocarcinoma (PAAD, *n* = 184), pheochromocytoma and paraganglioma (PCPG, *n* = 162), prostate adenocarcinoma (PRAD, *n* = 492), sarcoma (SARC, *n* = 257), skin cutaneous melanoma (SKCM, *n* = 367), stomach adenocarcinoma (STAD, *n* = 441), testicular germ cell tumor (TGCT, *n* = 150), thyroid carcinoma (THCA, *n* = 499), thymoma (THYM, *n* = 123), uterine corpus endometrial carcinoma (UCEC, *n* = 539), uterine carcinosarcoma (UCS, *n* = 56), and uveal melanoma (UVM, *n* = 80). Survival information (11) and RSEM-normalized gene-level data from cancers with *CCND1* amplification frequency ranked first to 10th were further downloaded. The data of CHOL were excluded for a limited number of samples (*n* = 36). Patients with *CCND1* amplification or neutral phenotypes were further analyzed. Patients with overall survival (OS) more than 10 days and gene-level data were enrolled as the TCGA pan-cancer cohort (*n* = 2,633). The TCGA pan-cancer cohort consisted of BLCA (*n* = 247), BRCA (*n* = 714), ESCA (*n* = 122), HNSCC (*n* = 359), LIHC (*n* = 277), LUSC (*n* = 292), OV (*n* = 145), SKCM (*n* = 203), and STAD (*n* = 274). OS was defined from the date of initial pathologic diagnosis.

We reviewed the CNA data from 10,109 solid tumor tissue samples from 10,109 patients in the MSKCC database who were enrolled as the MSKCC cohort (12). Survival information from cancers with *CCND1* amplification frequency ranked first to 10th was further downloaded. These patients with an OS of more than 10 days were enrolled as the MSKCC pan-cancer cohort (*n* = 3,629). Their OS was defined from the date of initial pathologic diagnosis. A total of 1,105 patients treated at MSKCC who had received at least one dose of ICIs had an OS defined from the date of first infusion of any ICI and were enrolled as the MSKCC-IO cohort (13).

To explore the association between *CCND1* amplification and the clinical outcomes of ICIs, we included CNA and clinical data from four clinical cohorts treated with ICIs. The first cohort consisted of 72 patients with melanoma treated with anti-cytotoxic T lymphocyte-associated protein 4 (CTLA-4) therapy (Allen cohort) (14). The second pooled cohort consisted of 464 melanoma patients treated with ICIs (Robert cohort treated with anti-PD-1/L1 or anti-CTLA-4 or combination therapy, Allen and Snyder cohorts treated with anti-CTLA-4 therapy, David cohort treated with anti-PD-1 therapy) (13–16).

### Database Analysis for *CCND1* and Tumor Mutational Burden

We analyzed the CNA in the TCGA and MSKCC cohorts. The CN changes, including putative biallelic CNA (+2) or putative biallelic neutral (0), identified using the GISTIC2 (17) algorithm in the TCGA samples and those using the FACETS (10) algorithm in the MSKCC samples, were the focus of our study. For the assessment of tumor mutational burden (TMB), the total number of somatic mutations identified was normalized to the exonic coverage of the respective MSK-IMPACT panel in megabases (13). Mutations in driver oncogenes were not excluded from the analysis (13). For each histology, cases in the top 20th, 40th, 60th, and 80th percentile of TMB were identified (13). Cases in the top 20th percentile of TMB within each histology were enrolled as the TMB-High group (*n* = 268).

### Tumor Purity Estimate and Infiltration Levels of Immune Cells

To investigate the immune infiltration status of the tumors, we computed tumor purity using the ESTIMATE (18) (Estimation of STromal and Immune cells in MAlignant Tumor tissues using Expression data) method to analyze immune components and overall stroma in the TCGA pan-cancer cohort. To measure the relative levels of tumor-infiltrating lymphocyte subsets, we then employed gene expression-based computational methods to profile the infiltration levels of 25 immune cell populations in the TCGA pan-cancer cohort. Among the 25 immune cell populations, 24 immune cell populations were calculated using methods named Immune Infiltration Score (IIS) and T cell Infiltration Score (TIS), while infiltration of myeloid-derived suppressor cells (MDSCs) was calculated utilizing an algorithm named Tumor Immune Dysfunction and Exclusion (TIDE) (19, 20).

### Gene Set Enrichment Analysis

Based on the hallmark gene sets (21), Gene Set Enrichment Analysis (GSEA) software version 3.0 (Broad Institute) (22) was used to identify the different regulated pathways between the *CCND1* amplification and neutral groups in the TCGA pan-cancer cohort (|NES| > 1, NOM *P*-value <0.10, FDR q-value <0.25). For significantly enriched pathways in the amplification group, single-sample gene set enrichment analysis (ssGSEA) was used to calculate the enrichment score in individual samples. The rank-sum test was performed to evaluate the statistical difference.

### Statistical Methods

Differences between the two groups were examined by two-tailed, unpaired *t*-test. Kaplan–Meier survival and multivariate Cox regression analyses were used to analyze associations between *CCND1* status and survival. Log-rank tests were used to determine significant differences of survival curves stratified by TMB. Statistical analyses were performed using SPSS statistical software version 23.0 (SPSS) and Prism analysis and graphic software version 8.0.1 (GraphPad). A two-sided *P*-value of <0.05 was considered statistically significant.

## Result

### Distribution and Clinical Implication of the *CCND1* Amplification Profile Landscape

**Figure 1:**
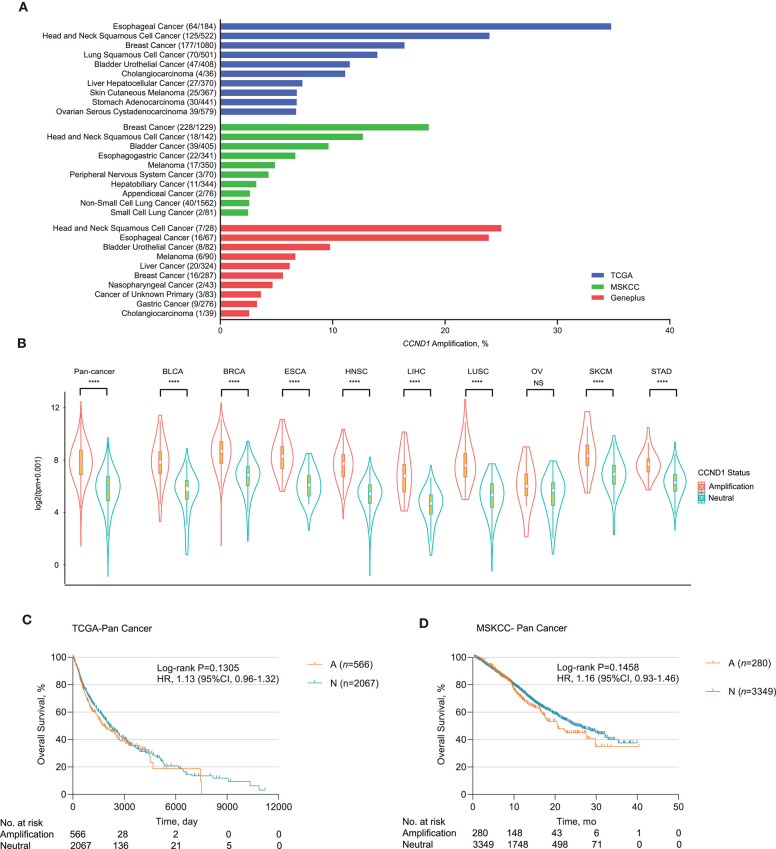
Profile of *cyclin D1 (CCND1)* amplification and association with prognosis. **(A)** Distribution of the top 10 cancer types with the frequency of *CCND1* amplification in The Cancer Genome Atlas (TCGA) (*n* = 10,606), Memorial Sloan Kettering Cancer Center (MSKCC) (*n* = 10,109), and Geneplus (*n* = 6,536) databases. Cancers were sorted according to the frequency of *CCND1* amplification. **(B)** The gene expression profile of *CCND1* between the amplification group and the neutral group in the TCGA pan-cancer cohort (*n* = 2,633). The white dot represents the median value. The bottom and top of the violins are the 25th and 75th percentiles (interquartile range). Differences between the two groups were evaluated by unpaired *t*-tests. ns *P* ≥ 0.05; *P < 0.05; **P < 1 × 10^−2^; ***P < 1 × 10^−3^; ****P < 1 × 10^−4^. **(C)** Kaplan–Meier survival analysis of overall survival (OS) comparing the *CCND1* amplification and neutral groups in patients in the TCGA pan-cancer cohort (*n* = 2,633). **(D)** Kaplan–Meier survival analysis of OS comparing the *CCND1* amplification and neutral groups in patients in the MSKCC pan-cancer cohort (*n* = 3,629).

We analyzed *CCND1* amplification of 6,536 patients from the Geneplus 1,021 panel in a Chinese population and found that HNSCC had a high *CCND1* amplification in 25.00% of the cases (7/28), followed by ESCA in 23.88% (16/67), BLCA in 9.76% (8/82), and melanoma in 6.67% of cases (6/90) (Figure 1A and Table S2). Comparison of *CCND1* amplification in 10,606 patients from the TCGA database and 10,109 patients from the MSKCC database revealed that ESCA and breast carcinoma had the highest incidence in the TCGA patients at 34.78% (64/184) and MSKCC patients at 18.55% (228/1,229) databases, respectively (Figure 1A). Gene expression analysis from the TCGA database showed that *CCND1* amplification was significantly related to the upregulation of mRNA expression of *CCND1* across the top nine cancer types (TCGA pan-cancer: cancers with *CCND1* amplification frequency ranked first to 10th; CHOL was excluded because of the limited number of samples) (Figure 1B).

Next, we examined the association of *CCND1* amplification with clinical outcome for pan-cancer in the TCGA and MSKCC databases. Kaplan–Meier survival analysis showed that *CCND1* amplification was not associated with median OS for pan-cancer in the TCGA database. The median OS for the *CCND1* amplification and *CCND1* neutral groups was 1,838.0 and 2,133.0 days, respectively [*P* = 0.1305, HR 1.13 (95% CI 0.96–1.32); Figure 1C]. In the MSKCC database, the median OS for the *CCND1* amplification and *CCND1* neutral groups was 20.6 and 25.4 months, respectively [*P* = 0.1458, HR 1.16 (95% CI 0.93–1.46); Figure 1D]. We then further investigated the role of *CCND1* amplification in specific cancer types. We did find a significantly decreased OS for HNSCC in the TCGA database. The median OS for the *CCND1* amplification and *CCND1* neutral groups was 1,079.0 and 2,002.0 days, respectively [*P* = 0.0125, HR 1.51 (95% CI 1.07–2.11); Figure S1A]. For melanoma in the MSKCC database, the median OS for the *CCND1* amplification and *CCND1* neutral groups was 13.5 months and not reached [*P* = 0.0139, HR 2.56 (95% CI 0.79–8.29); Figure S1B].

### *CCND1* Amplification Associated With Poor Prognosis in Patients Who Received Immune Checkpoint Inhibitors

**Figure 2:**
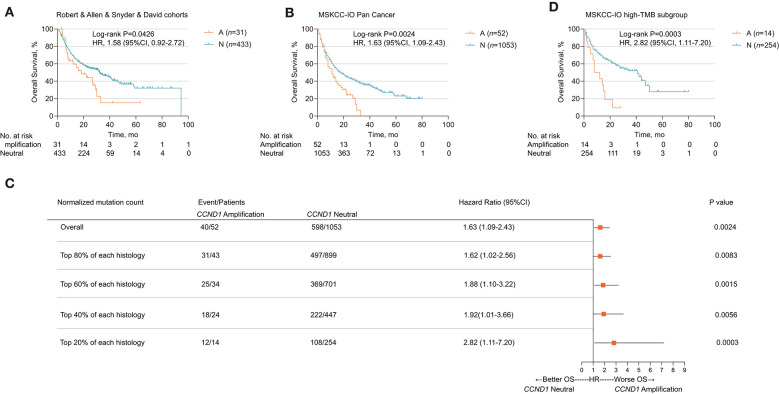
Association of *cyclin D1 (CCND1)* amplification with prognosis in the Memorial Sloan Kettering Cancer Center (MSKCC)-IO cohort. **(A)** Kaplan–Meier survival analysis of overall survival (OS) comparing the *CCND1* amplification and neutral groups in patients with melanoma treated with immune checkpoint inhibitors (ICIs) from the Robert, Allen, Snyder, and David cohorts (*n* = 464). **(B)** Kaplan–Meier survival analysis of OS comparing the *CCND1* amplification and neutral groups in patients with solid tumors treated with ICIs in the MSKCC-IO pan-cancer cohort (*n* = 1,105). **(C)** Hazard ratios of *CCND1* status across patients with different levels of tumor mutational burden (TMB) in the MSKCC-IO pan-cancer cohort (*n* = 1,105). **(D)** Kaplan–Meier survival analysis of OS comparing the *CCND1* amplification and neutral groups in patients with solid tumors treated with ICIs identified with top 20% TMB within each histology in the MSKCC-IO cohort (*n* = 268).

We further explored the relationship between *CCND1* amplification and the clinical outcomes of ICIs. Publicly available datasets were utilized for this analysis, including four melanoma clinical cohorts. The Allen cohort (14) included 72 patients with melanoma treated with anti-CTLA-4, among whom six patients were classified as *CCND1* amplification; their disease-free survival (DFS) was inferior to that of 66 patients with the neutral phenotype [2.667 vs. 3.233 months, *P* = 0.1196, HR 2.001 (95% CI 0.5786–6.922); Figure S1C]. Then, we performed a pooled analysis on four melanoma cohorts treated with ICIs (the Robert cohort treated with anti-PD-1/L1 or anti-CTLA-4 or combination therapy, the Allen and Snyder cohorts treated with anti-CTLA-4 therapy, and the David cohort treated with anti-PD-1 therapy, *n* = 464) (13–16). A total of 31 patients were classified as having *CCND1* amplification, and their median OS was significantly shorter than 433 patients with a *CCND1* neutral status [18.51 vs. 32.27 months, *P* = 0.0426, HR 1.58 (95% CI 0.9163–2.724); Figure 2A].

Based on the impact of *CCND1* amplification as a negative prognostic factor for efficacy of ICIs in melanoma, we further investigated its role in patients with a solid tumor. To validate *CCND1* amplification as a clinical factor associated with poor prognosis in patients with solid tumors treated with ICIs, we performed three analyses. First, a total of 1,105 patients with a variety of cancer types who had received MSKCC-IMPACT testing and at least one dose of ICIs were evaluated and named the MSKCC-IO cohort (13). Fifty-two patients with *CCND1* amplification were identified comprising of 14 melanomas, 11 head and neck carcinomas (HNCs), 11 bladder carcinomas, eight non-small-cell lung carcinomas, five breast carcinomas, three esophagogastric carcinomas, and one glioma (Table S3). Across the entire cohort, *CCND1* amplification was associated with a decreased OS. The median OS for the *CCND1* amplification and *CCND1* neutral groups was 11.0 and 18.0 months, respectively [*P* = 0.0024, HR 1.63 (95% CI 1.09–2.43); Figure 2B]. We performed a stratified analysis with the melanoma (*n* = 231) and bladder carcinoma patients (*n* = 111) and observed a similar association between *CCND1* amplification with a shorter OS. In melanoma (*n* = 231), the median OS for the *CCND1* amplification and *CCND1* neutral groups was 22.0 and 42.0 months [*P* = 0.0029, HR 2.48 (95% CI 0.99–6.23); Figure S1D]. In bladder carcinoma (*n* = 111), the median OS for the *CCND1* amplification and *CCND1* neutral groups was 8.0 and 16.0 months, respectively [*P* = 0.0244, HR 2.17 (95% CI 0.83–5.66); Figure S1E].

Recent studies have shown that a high level of TMB associates with improved survival in patients receiving ICIs across a wide variety of cancer types (13, 23). We therefore explored the relationship between *CCND1* amplification and TMB, and their interaction related to ICI efficacy. We compared the TMB between the *CCND1* amplification group and the *CCND1* neutral group in the MSKCC-IO cohort and found no difference between the two groups. The median TMB for the *CCND1* amplification and *CCND1* neutral groups was 6.79 vs. 5.90 (*P* = 0.46; Figure S2). We then prepared a stratified analysis of the MSKCC IO-cohort by the percentile of the TMB subgroup. A clear profile demonstrated that the *CCND1* amplification patients did not benefit from ICIs regardless of TMB status (Figure 2C). Of note, according to a study by Robert M. Samstein et al. (13), in patients treated with ICIs, there is a significant association between a high level of TMB and a better OS. But in our stratified analysis, in spite of a high level of TMB, patients with *CCND1* amplification have a significantly decreased median OS [10.0 vs. 41.0 months, HR 2.82 (95% CI 1.11–7.20), *P* = 0.0003; Figure 2D]. Finally, a multivariable analysis using Cox proportional-hazards regression demonstrated that *CCND1* amplification was significantly associated with a shorter median OS [HR 1.60 (95% CI 1.16–2.21), *P* = 0.0040], with adjustment for TMB, cancer type, age, drug class of ICI, and the year of ICI start (Table S4).

Furthermore, we compared the OS in *CCND1* amplification patients who received or did not receive ICIs. In the MSKCC cohort, data from Zehir et al. (12) reported 319 patients with *CCND1* amplification and 46 cases received ICIs. We found that the ICI group has a shorter median OS than the non-ICI group [17.5 vs. 22.6 months, HR 1.258 (95% CI 0.75–2.10), *P* = 0.3411; Figure S3]. Taken together, this implies that ICIs would not be useful for treating the *CCND1* amplification population.

### *CCND1* Amplification Is Significantly Associated With a Signature of Tumor Immunosuppression and Immune Cell Exclusion

**Figure 3:**
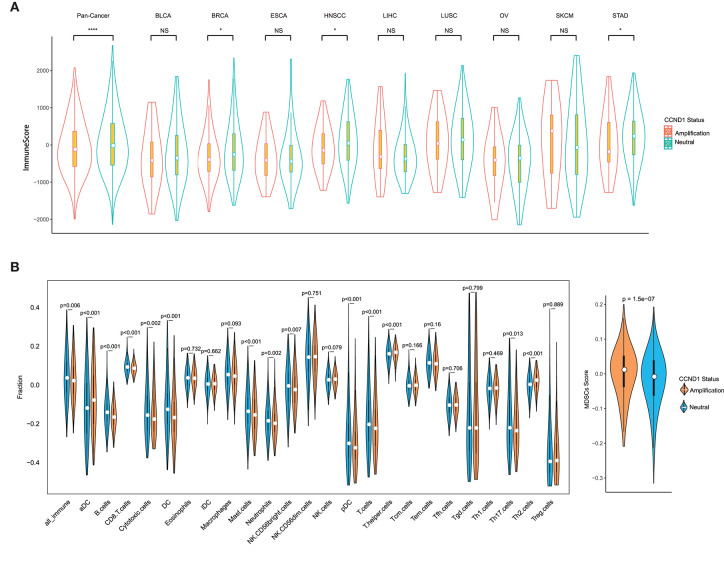
Tumor purity estimate and infiltration levels of immune cells in The Cancer Genome Atlas (TCGA) pan-cancer cohort. **(A)** The ESTIMATE (Estimation of STromal and Immune cells in MAlignant Tumor tissues using Expression data) score of cancers between the *cyclin D1 (CCND1)* amplification group and the neutral group in TCGA pan-cancer cohort (*n* = 2,633). The white dot represents the median value. The bottom and top of the violins are the 25th and 75th percentiles (interquartile range). Differences between the two groups were evaluated by unpaired *t*-tests. ns *P* ≥ 0.05; *P < 0.05; **P < 1 × 10^−2^; ***P < 1 × 10^−3^; ****P < 1 × 10^−4^. **(B)** The measurement of the infiltration levels of 25 immune cell populations between the *CCND1* amplification group and the neutral group in the TCGA pan-cancer cohort (*n* = 2,633). The median white dot represents the median value, while the upper and lower represent the minimum and maximum values. Differences between the two groups were evaluated by unpaired *t*-tests.

Remarkable associations have been observed between the presence of immune cells, especially with tumor-specific T cell infiltration, and/or a T cell-associated inflammatory gene expression signature and the response to ICIs (23, 24). To assess the relationship between *CCND1* amplification and the landscape of immune cell infiltration, we used an algorithm called ESTIMATE (18) to analyze the infiltrating fraction of stromal and immune cells in tumor samples from the TCGA pan-cancer cohort (*n* = 2,633). We found that the median ESTIMATE score in the *CCND1* amplification group was significantly inferior to that in the neutral group (−849.10 vs. −696.23, *P* = 0.0051; Figure 3A). The analysis in nine individual cancer types showed that most cancer types exhibited a similar trend with the exception of melanoma, ESCA, and liver hepatocellular carcinoma. For example, in breast cancer, the *CCND1* amplification group showed a lower median ESTIMATE score (−669.12 vs. −245.99, *P* = 0.0130; Figure 3A). In HNSCC, the *CCND1* amplification group also exhibited a lower median ESTIMATE score (−849.10 vs. −716.92, *P* = 0.0190; Figure 3A).

To further characterize the levels of distinct immune cell subsets and the signals that are chemotactic for them, we employed a gene expression-based computational method to dissect infiltration of 25 immune cell subsets in the TCGA pan-cancer cohort (19, 20). Strikingly, the analysis of the transcriptomes revealed that, compared with the *CCND1* neutral group, the *CCND1* amplification group had a decrease in the median value of CD8^+^ T cells (0.0854 vs. 0.0920, *P* < 0.001), cytotoxic cells (−0.1772 vs. −0.1564, *P* = 0.0020), dendritic cells (DCs) (−0.1692 vs. −0.1266, *P* < 0.001), plasmacytoid DC cells (pDCs) (−0.3249 vs. −0.3020, *P* < 0.001), and B cells (−0.1666 vs. −0.1419, *P* < 0.001). There was an increase in T helper cells (0.1677 vs. 0.1603, *P* < 0.001), regulatory T (Treg) cells (0.0240 vs. −0.3954, *P* = 0.8890), activated dendritic cell (−0.0787 vs. 0.0356, *P* < 0.001), and MDSCs (0.0115 vs. −0.0087, *P* < 0.001) (Figure 3B).

In the subtype analysis of 25 immune cell components in nine tumor types, we found that various degrees of tumor microenvironmental immunosuppression occurs (Figure S4). For example, the median values of B cells (−0.1870 vs. −0.1691, *P* < 0.001), T cells (−0.2160 vs. −0.1935, *P* = 0.0090), CD8^+^ T cells (0.0914 vs. 0.1009, *P* < 0.001), and DC cells (−0.1810 vs. −0.1465, *P* = 0.0170) were significantly attenuated in the *CCND1* amplification group in breast cancer, while Th2 cells (0.05419 vs. 0.0148, *P* < 0.001) and MDSCs (0.0051 vs. −0.0198, *P* = 0.0094) appear upregulated (Figure S4). The signature of immune cell subsets in HNSCC showed a dramatic decrease in median values of cytotoxic cells (−0.1418 vs. −0.0970, *P* = 0.0030), T cells (−0.2357 vs. −0.2056, *P* = 0.0010), CD8^+^ T cells (0.0730 vs. 0.0761, *P* = 0.1310), DC cells (−0.2267 vs. −0.1796, *P* < 0.001), and B cells (−0.1676 vs. −0.1373, *P* < 0.001), while MDSCs (0.0250 vs. −0.0058, *P* < 0.001) (Figure S4).

### Multiple Aggressive, Immunosuppressive, and Angiogenic Hallmarks Related With *CCND1* Amplification

**Figure 4:**
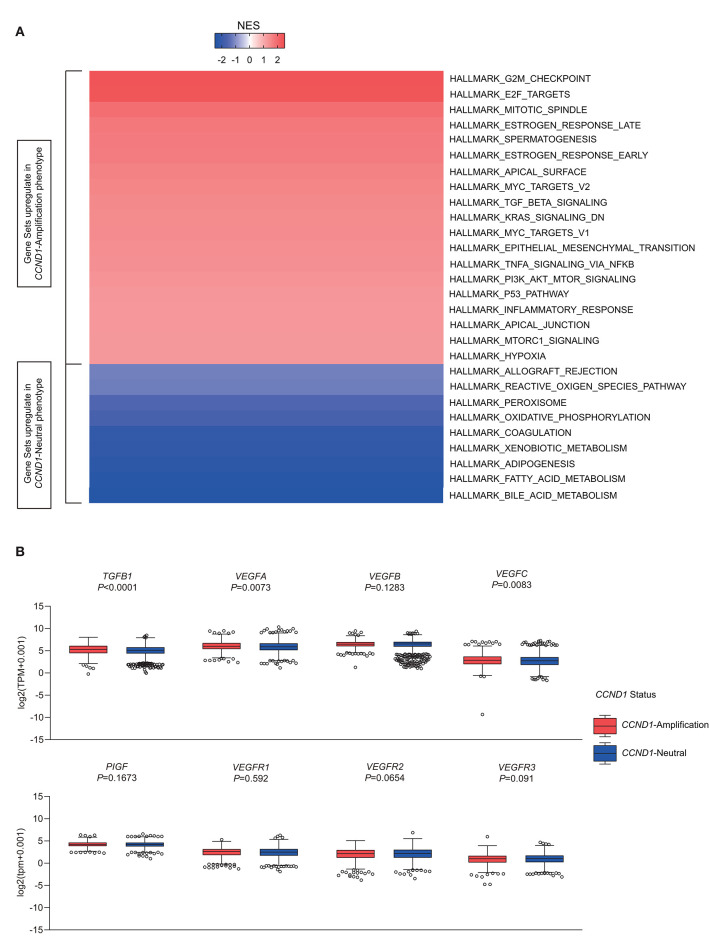
Identification of hallmarks associated with *cyclin D1 (CCND1)* amplification in The Cancer Genome Atlas (TCGA) pan-cancer cohort (*n* = 2,633). **(A)** Different upregulation of inflammatory pathways among the *CCND1* amplification group and the *CCND1* neutral group in the TCGA pan-cancer cohort. The result is expressed according to the normalized enrichment score (NES). **(B)** The box and whiskers plots depict differences in transcript-level changes of *transforming growth factor (TGF)B1, vascular endothelial growth factor (VEGF)A–C, placental growth factor (PIGF)*, and *VEGF receptor (VEGFR)1–3* between the *CCND1* amplification group and the neutral group in the TCGA pan-cancer cohort. Within each group, the scattered dots represent gene values, and the thick line represents the median value. The bottom and top of the boxes are the 25th and 75th percentiles. Differences between the two groups were determined by unpaired *t*-tests.

To investigate signaling pathways activated for *CCND1* amplification tumors, we performed GSEA comparing the *CCND1* amplification group and the *CCND1* neutral group in the TCGA pan-cancer cohort. GSEA revealed significant differences (false discovery rate-q ≤ 0.25) in the enrichment of the Hallmark database (Figure 4A). Notably, gene sets related to epithelial mesenchymal transition, mitotic spindle, myc targets, transforming growth factor (TGF)-β signaling, KRAS signaling, tumor necrosis factor (TNF)-α signaling via nuclear factor (NF)-κB, phosphoinositide 3-kinase (PI3K)/AKT/mammalian target of rapamycin (mTOR) signaling, p53 pathway, mTOR complex 1 (MTORC1) signaling, and the hypoxia signaling pathways were differentially upregulated in the *CCND1* amplification phenotype (Figure 4A). Interestingly, in HNSCC, we also found angiogenesis in the *CCND1* amplification phenotype, while the activities of the interferon-α/β response, interleukin (IL)6-Janus kinase (JAK)-signal transducer and activator of transcription (STAT)3 signaling, and Wnt-β catenin signaling pathways were increased in the neutral phenotype (Figure S5).

Since TGF-β (encoded by *TGFB1*), hypoxia-inducible factor (HIF)1A (encoded by *HIF1A*), vascular endothelial growth factors (VEGFs) [encoded by *VEGFA-C, placental growth factor (PIGF), VEGF receptor (VEGFR)1-3*], angiopoietin growth factors (encoded by *ANGPT1-2*), MET (encoded by *MET*), hepatocyte growth factor (HGF) (encoded by *HGF*), platelet-derived growth factors (PDGFs) (encoded by *PDGFA-D, PDGFRA-B, PDGFRL*), fibroblast growth factor (FGF)2 (encoded by *FGF2*), FGFR2 (encoded by *FGFR2*), and adhesion molecules [encoded by *intercellular adhesion molecule* (*ICAM)1, vascular cell adhesion molecule (VCAM)1, CD34*] might modify the cancer-related immune microenvironment and decrease the efficacy of immunotherapies (25), we analyzed the RNA-Seq data in TCGA focusing on single genes including *TGFB1, HIF1A, MET, HGF*, adhesion molecules, angiopoietin growth factors, PDGF family, and VEGFs family (Table S5). In the TME of TCGA pan-cancer cohort, *CCND1* amplification showed a statistically significant correlation with high expression of *TGFB1* (5.293 vs. 5.108, *P* < 0.0001), *VEGFA* (5.992 vs. 5.854, *P* = 0.0073), *VEGFB* (6.458 vs. 6.483, *P* = 0.1283), *ICAM1* (4.721 vs. 4.896, *P* = 0.2596), and *HIF1A* (6.028 vs. 5.761, *P* < 0.0001) in the TME (Figure 4B and Figure S6). Previous studies had revealed that *VEGFA* has direct or indirect effects on components of the immune system, including suppressing DC maturation and CD8^+^ T cell proliferation (25) and affecting *ICAM1* to suppress NK cell and T cell trafficking (25), resulting in immunosuppressive outcomes. Another study showed that cyclin D1 (encoded by *CCND1*) may play a key role in the maintenance of VEGFs, and antisense to cyclin D1 could be useful for targeting both cancer cells and blood vessels in tumors (26). Above all, we deduced that anti-VEGFs/VEGFRs may potentially reverse the *CCND1* amplification that is associated with resistance to ICIs.

## Discussion

In this study, we comprehensively described the *CCND1* amplification profile in the TCGA and MSKCC databases and in a Chinese population in the Geneplus cohort. We found that *CCND1* amplification can hinder not only the natural host immune response but also the efficacy of ICIs. A *CCND1* amplification may potentially identify a patient population that will not benefit from ICIs irrespective of TMB status.

The *CCND1* located on human chromosome 11q13.3 is considered an oncogene, and it increases cell proliferation, growth, angiogenesis, and resistance to chemotherapy and radiotherapy (3, 4). To our knowledge, our results are the first to reveal that a *CCND1* amplification may significantly correlate with tumorigenesis and attenuation of various types of effector immune cells in the TME, including cytotoxic cells, T cells, CD8^+^ T cells, DC cells, and B cells, and upregulation of Treg cells and MDSCs. Oncogenes such as *PDGFA-D, FGF2, HGF*, and *MET* are significantly overexpressed in the *CCND1* amplification group, promoting the development and progression of tumors. Previous studies have shown the role of the cytokine TGF-β, promoting immunosuppression in the TME (2, 20, 27, 28). In our analysis of the TCGA pan-cancer cohort, *CCND1* amplification showed a statistically significant correlation with high mRNA expression of *TGFB1*. More importantly, further study showed significant upregulation of mRNA expression of *VEGFA*, another known factor inducing tumor immune escape and immunotherapy resistance (25), associated with the *CCND1* amplification phenotype.

From the survival analysis in TCGA and MSKCC public databases, we found no significant correlation between *CCND1* amplification with prognosis in the pan-cancer group. There are some reasons to interpret this result. Firstly, the source of samples enrolled in the TCGA and MSKCC databases were diverse, and the clinical pathological characters of patients were complicated. Hence, the differences between cancer types must be taken into account. Secondly, in previous studies, methods such as Fluorescence *in situ* Hybridization (FISH), Chromogenic *in situ* Hybridization (CISH) or Reverse Transcription-Polymerase Chain Reaction (RT-PCR) were used to detect amplification of genes. Some studies used immunohistochemistry to stain cyclin D1. But there was no consistency on the definition of amplification of genes or the high- or low-expression level of cyclin D1 in various cancer types or within the same cancer type. Here, in our study, we used sequencing of genes by a CNV technique to detect amplification of genes. Meanwhile, the analysis of the transcriptome showed that the amplification of *CCND1* was strongly correlated with higher expression level of mRNA. This also increases the credibility of the results and unifies the consistency of the detection. Thirdly, according to our investigation, activations of a variety of oncogenes and deactivations of tumor suppressor genes were observed along with the amplification of CCND1 in different cancer types. Therefore, when the sample is enlarged and after balancing different tumor types, the value of *CCND1*'s impact on prognosis may be weakened.

Nevertheless, the *CCND1* amplification is a potential predictive biomarker for the use of ICIs in patients with solid tumors. In the melanoma pooled cohort, the median OS was shorter in the *CCND1* amplification subgroup. The survival analysis in the MSKCC-IO cohort further verified the negative impact of *CCND1* amplification on the efficacy of ICIs. Strikingly, by comparing *CCND1* amplification with TMB in patients with solid tumors from the MSKCC-IO cohort, we found that the association between *CCND1* amplification and a worse clinical outcome was more distinct in TMB-high patients. This indicates that ICIs may not be useful, and even harmful, to patients with *CCND1* amplification. We propose three hypotheses to explain the impairment for ICI efficacy. First, various types of effector immune cell exclusion and immunosuppression in the TME were found in tumors with *CCND1* amplification. Second, *CCND1* amplification results in high mRNA expression of *TGFB1, VEGFA*, and *HIF1A*; these molecules have direct or indirect negative effects on components of the immune system. Finally, some oncogene pathways are activated in *CCND1* amplification tumor that may lead to acceleration of tumor growth. Recently, a study reported on five patients experiencing hyper-progression who had NGS performed on pretreatment tumor tissue, and it confirmed CNAs in *MDM2*/*MDM4, epidermal growth factor receptor (EGFR)*, and several genes located on 11q13 associated with hyper-progression (29). So, these results will establish an important foundation for screening patients who might not benefit from ICI therapy.

Considering the immunosuppression in the TME and overexpression of various oncogenes caused by *CCND1* amplification, patients with such features should avoid ICI monotherapy. Multi-combination strategies including anti-angiogenesis agents or anti-TGF-β agents may eliminate the latent immunosuppressive factors in the TME and reverse the resistance to ICIs.

Our study has some limitations. When *CCND1* amplification is included in the interpretation of cancer prognosis, issues such as tumor type, standardization of detection, and accompanying gene mutation status should be fully considered. The small number of *CCND1* amplification tumors and the rarity of the event suggest that further additional data are warranted. The analysis of additional trials and cohorts will improve the precision of our estimates and the robustness of our findings. Our study is a preliminary investigation mainly focused on the predictive function of *CCND1* amplification in the tumor microenvironment in the aspect of genome and transcriptome. The full implication of *CCND1* amplification remains elusive and requires in-depth studies. Experiments to investigate the direct mechanism of *CCND1* amplification and primary immune resistance should be performed.

These findings indicate that *CCND1* amplification may be a key point related to immunosuppression in the TME and multiple malignancy hallmark; it may be a common mechanism of resistance to ICIs.

## Data Availability Statement

The raw data supporting the conclusions of this article will be made available by the authors, without undue reservation, to any qualified researcher.

## Ethics Statement

The studies involving human participants were reviewed and approved by Ethics Committee of Fujian Provincial Cancer Hospital. Written informed consent to participate in this study was provided by the participants' legal guardian/next of kin. Written informed consent was obtained from the individual(s) for the publication of any potentially identifiable images or data included in this article.

## Author Contributions

YC, YH, XG, and YL were responsible for conducting the study, under the supervision of CC and XY, and contributed to the study design. YC, YH, XG, YL, JL, LChe, YX, GC, YG, LP, XX, ZG, JP, XY, LCha, and CC acquired the data. YC, YH, XG, YL, YX, YG, XX, ZG, JP, XY, and CC performed the data analysis and interpretation. YC, YH, XG, YL, and CC wrote and revised the manuscript. All authors contributed to the article and approved the submitted version.

## Conflict of Interest

XG, LC, YG, XX, YX, and XY are current employees of Geneplus-Beijing. YG, XX, and XY hold leadership positions and stocks of Geneplus-Beijing. The remaining authors declare that the research was conducted in the absence of any commercial or financial relationships that could be construed as a potential conflict of interest.

## Abbreviations

*CCND1*: cyclin D1
ICIs: immune checkpoint inhibitors
NGS: next-generation sequencing
TCGA: The Cancer Genome Atlas
MSKCC: Memorial Sloan Kettering Cancer Center
OS: overall survival
TMB: tumor mutational burden
TME: tumor microenvironment
PD-1: programmed cell death 1
PD-L1: programmed cell death ligand 1
HNSCC: head and neck squamous cell carcinoma
CNA: copy number alteration
BLCA: bladder urothelial carcinoma
BRCA: breast invasive carcinoma
CHOL: cholangiocarcinoma
ESCA: esophageal carcinoma
LIHC: liver hepatocellular carcinoma
LUSC: lung squamous cell carcinoma
OV: ovarian serous cystadenocarcinoma
SKCM: skin cutaneous melanoma
STAD: stomach adenocarcinoma
MDSCs: myeloid-derived suppressor cells
GSEA: gene set enrichment analysis
CTLA4: cytotoxic T lymphocyte-associated protein 4
DFS: disease-free survival
DCs: dendritic cells.
